# Continuous-wave GaAs/AlGaAs quantum cascade laser at 5.7 THz

**DOI:** 10.1515/nanoph-2023-0726

**Published:** 2024-01-16

**Authors:** Mohammad Shahili, Sadhvikas J. Addamane, Anthony D. Kim, Christopher A. Curwen, Jonathan H. Kawamura, Benjamin S. Williams

**Affiliations:** Department of Electrical and Computer Engineering, University of California, Los Angeles, CA 90095, USA; Sandia National Laboratories, Center of Integrated Nanotechnologies, MS 1303, Albuquerque, NM 87185, USA; Jet Propulsion Laboratory, California Institute of Technology, Pasadena, CA 91109, USA

**Keywords:** quantum cascade laser, terahertz, *Reststrahlen* band, gallium arsenide, nonequilibrium Green’s function

## Abstract

Design strategies for improving terahertz (THz) quantum cascade lasers (QCLs) in the 5–6 THz range are investigated numerically and experimentally, with the goal of overcoming the degradation in performance that occurs as the laser frequency approaches the *Reststrahlen* band. Two designs aimed at 5.4 THz were selected: one optimized for lower power dissipation and one optimized for better temperature performance. The active regions exhibited broadband gain, with the strongest modes lasing in the 5.3–5.6 THz range, but with other various modes observed ranging from 4.76 to 6.03 THz. Pulsed and continuous-wave (cw) operation is observed up to temperatures of 117 K and 68 K, respectively. In cw mode, the ridge laser has modes up to 5.71 THz – the highest reported frequency for a THz QCL in cw mode. The waveguide loss associated with the doped contact layers and metallization is identified as a critical limitation to performance above 5 THz.

## 1 Introduction

Terahertz (THz) quantum cascade lasers (QCLs) have an important application as sources for high-resolution spectroscopy of rotational transitions in polar molecules and fine structure lines of selected atomic species. Specifically, they can be used as heterodyne local oscillators to pump Schottky diode mixers or arrays of superconducting NbN and MgB_2_ mixers in astrophysical observations of the interstellar medium and planetary atmospheres [1]. Below 3 THz, Schottky diode frequency multiplier chains are the standard source for this application; however, their available power drops rapidly for higher frequencies. For this reason, QC-lasers have found a role on two recent heterodyne instruments – the upGREAT instrument on the SOFIA airborne observatory and the GUSTO ultra-long duration balloon observatory – which have targeted the neutral oxygen line [OI] at 4.74 THz [2], [3]. Indeed, THz QCLs provide milliwatt to tens-of-milliwatt levels of output power, which makes them appealing for pumping next generation heterodyne instruments with (many) tens of pixels. However, there are other compelling spectral lines above 5 THz that can be exploited, such as the elemental sulfur [SI] (5.32 THz) and iron [FeI] (5.52 THz), and doubly ionized nitrogen [NIII] (5.23 THz), oxygen [OIII] (5.79 THz), and iron [FeIII] (5.8 THz) [4]. Yet, there has been no demonstration of continuous-wave (cw) operation in a QCL above 5.26 THz, which is a necessary requirement for a local oscillator. The challenge in making THz QCLs above 5 THz lies in increased THz optical loss as well as a reduction in the intersubband (ISB) gain. Both effects are related to the proximity of the operating frequency to the *Reststrahlen* band of GaAs (8–9 THz) associated with optical phonon resonances, which makes the material highly absorptive and reflective [5], [6]. The main contributing factors to the waveguide loss above 5 THz are the increased losses from the GaAs phonons, the heavily doped contact layers, and the ISB absorption within the active region. While the GaAs phonon losses are inherent to the material and cannot be avoided, the metal cladding can be optimized by using the right materials and thicknesses, heavily doped contact layers can often be removed entirely, and the ISB absorption losses can be mitigated through active region design. The gain degradation above 5 THz is a result of increased nonradiative, thermally activated scattering of upper-state carriers to both lower and parasitic levels. For example, the nonradiative scattering rate between the upper and lower radiative states can be approximated with the thermally activated expression,

(1) $$\tau_{54}^{-1}\approx W_{54}^{\text{hot}}\exp\left(\frac{E_{54}-E_{\text{LO}}}{k_{\text{B}}T}\right)$$

where $W_{54}^{\text{hot}}$ is the scattering rate when the carriers in the upper radiative subband (labeled 5) have sufficient in-plane energy to emit a longitudinal-optical (LO) phonon and relax to the lower state (labeled 4). As the THz QCL frequency increases beyond 5 THz (*E*_54_ > 20.7 meV), it approaches the LO phonon energy of GaAs (*E*_LO_ = 36 meV) and the activation energy (*E*_LO_ – *E*_54_) is reduced. So far, these effects have limited the pulsed operation of QCLs above 5 THz to a maximum operating frequency of 5.6 THz [7] and only at temperatures below 100 K [8]. Additionally, there has been only one demonstration of cw operation above 5 THz, which was at 5.26 THz with a *T*_max_ of 15 K [8].

In this work, we present strategies for optimizing the THz QCL active region and waveguide above 5 THz. We then demonstrate this improvement by growing and testing two devices – labeled D1 and D2 – that are designed for ∼5.4 THz operation. The first design D1 is an incrementally modified version of a previous design aimed at 4.7 THz (details in the Supplementary Material); the barrier thicknesses are the same, and the well widths have been changed slightly to scale the gain up to 5.4 THz. For the second design D2, however, we performed a systematic numerical modeling process to optimize the design for improved temperature performance. Finally, we present a brief discussion on the effectiveness of these strategies for cw mode of operation.

## 2 Active region design

**Figure 1:**
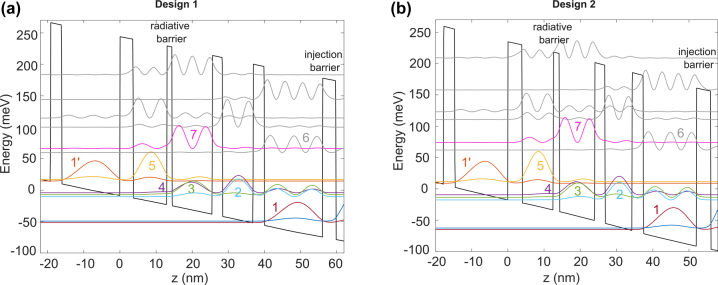
Conduction band diagram and wavefunction magnitude squared of a single module of (a) design 1 and (b) design 2. The key states are highlighted in color and labeled as 7, 6 (parasitic), 5 (upper state), 4, 3, 2 (lower states), and 1 (1′) (ground).

We base our active region design strategy around the hybrid bound-to-continuum/resonant-phonon (BTC-RP) scheme [9], with high Al_0.25_Ga_0.75_As barriers (∼250 meV band offset) to suppress over-the-barrier leakage [10]; two examples of this are shown in Figure 1. The upper and lower radiative states are labeled 5 and 4, respectively (although at some biases, there can also be a significant oscillator strength between level 5 and 3). Depopulation of the lower state(s) takes place through a combination of electronic scattering, tunneling, and finally fast LO-phonon scattering into the injector state 1. Also, of concern are states 6 and 7, which can act as a second thermally activated parasitic current channel. It is convenient in our following discussion to refer to the following simple relation for the peak ISB gain coefficient,

(2) $$g_{54}\propto \frac{J}{e}\frac{f_{54}}{\Delta \nu }\tau_{5}\left(1-\frac{\tau_{4}}{\tau_{54}}\right),$$

where *J* is the injection current density into the upper state, *e* is the electron charge, *f*_54_ is the transition oscillator strength, and Δ*ν* is the ISB transition linewidth. A similar expression for the ISB gain associated with transitions from 5 → 3 can be written if needed.

**Figure 2:**
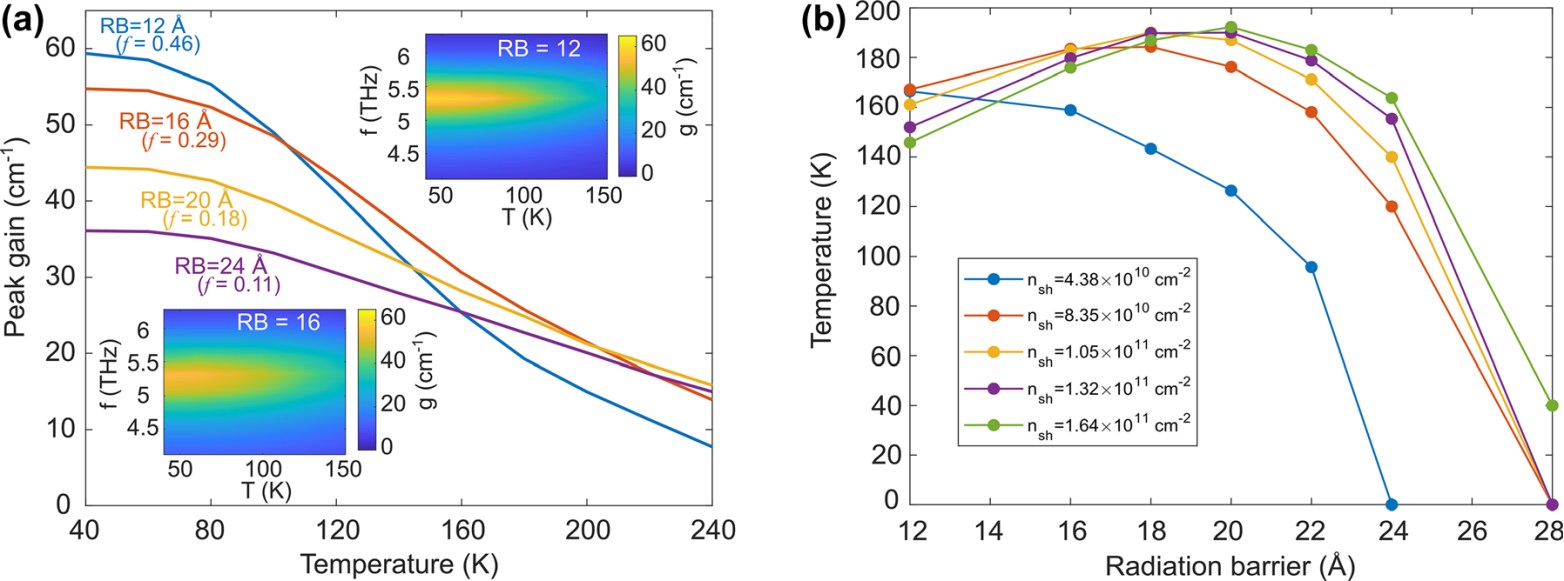
NEGF simulation of 5.4 THz QCLs with varying radiative barrier (RB) thicknesses. (a) Peak gain coefficient versus temperature for different RB thicknesses. The insets show the gain spectra for RBs 12 Å and 16 Å versus temperature. (b) Temperature at which gain coefficient = 25 cm^−1^ versus RB thicknesses for different sheet doping densities (*n*_sh_).

Our strategy for improving >5 THz performance has the following elements. First, we use the well-known principle that the upper state lifetime *τ*_5_ can be increased by reducing the wavefunction overlap between level 5 and 4 [11]–[14]; i.e., making the radiative transition more spatially diagonal reduces almost all scattering rates out of level 5, including *W*_54_^hot^ of Eq. (1). Increased diagonality, however, comes at the price of reduced oscillator strength *f*_54_, which in turn reduces *g*_54_. To quantify this effect, we performed a systematic numerical study using the nextnano nonequilibrium Green’s function (NEGF) simulation package [15] to plot the peak gain coefficient for a set of 5.4 THz active regions where the level of diagonality was varied by changing the radiative barrier (RB) thickness from 12 to 24 Å, as shown in Figure 2(a), while small changes to well thicknesses were made to maintain the same transition frequency. While the active regions with a thicker RB have lower gain at low temperatures compared to the designs with a thinner RB, their gain degrades more slowly with increasing temperature. This slower gain degradation is a clear indication of the decreased electron-optical-phonon scattering of the upper state electrons due to a smaller spatial overlap of upper and lower state carriers (see (1)). At low temperatures, the phonon scattering of electrons in the upper state is reduced, and the gain is higher for designs with a thinner RB due to a higher *f*_54_, as is also inferred from (2). The increased diagonality also results in a broader linewidth Δ*ν*, likely due to the increased effect of interface roughness scattering (see Figure 2(a) inset).

Second, we consider the use of higher doping levels – which results in a larger maximum injected current density *J*_max_ – to counter the reduced oscillator strength that accompanies the more diagonal transition [16]. Again, a set of NEGF numerical experiments is used to plot a nominal maximum operating temperature (*T*_max_) value (i.e., the temperature where the peak gain coefficient is reduced to equal a waveguide loss coefficient of 25 cm^−1^) versus RB thickness for varying sheet doping densities, as shown in Figure 2(b). The improvement in *T*_max_ is clearly seen for thick RBs (>14 Å) as the sheet doping density is increased. For very thick RBs (>20 Å), the *T*_max_ eventually drops as the oscillator strength becomes too small. For thin RBs (<14 Å), however, the increased sheet doping density seems to decrease *T*_max_. This finding contrasts with the previous investigations of this behavior using a rate-equation model [16], and the difference can be attributed to the increased electron temperature associated with larger current density as well as increased electron-impurity scattering, both of which increase nonradiative scattering from the upper state into both lower and parasitic states. Therefore, there is an optimal range of radiative barrier thicknesses (18–20 Å) where current density is not excessively high, and a diagonal, high-doped design leads to the maximum improvement in *T*_max_.

Third, in addition to using tall Al_0.25_Ga_0.75_As barriers, we have the option of generating designs with overall thinner GaAs wells. While this pushes all of the subband energies up in the band structure, the effect is more pronounced for the parasitic states 6 and 7, since they have the character of excited quantum well states, whose energies scale as the square of the quantum number. This will help bring the design closer to a clean 5-level system by reducing coupling to the higher-lying parasitic states [10], [17], [18]. Additionally, this has the effect of increasing the depopulation energy (*E*_21_) above that of the bulk GaAs LO phonon energy (36 meV). This is not believed to cause a large change in the depopulation rates [19]. However, it will have a significant benefit for QCLs >5 THz as the photon energy (*E*_54_ > 20.7 meV) approaches *E*_21_. Since the injector state 1 typically holds the majority of the electronic population even at design bias, there is a strong ISB absorption at the energy *E*_21_. By increasing *E*_21_, the loss associated with the wings of the ISB transition lineshape at *E*_54_ is effectively reduced.

**Figure 3:**
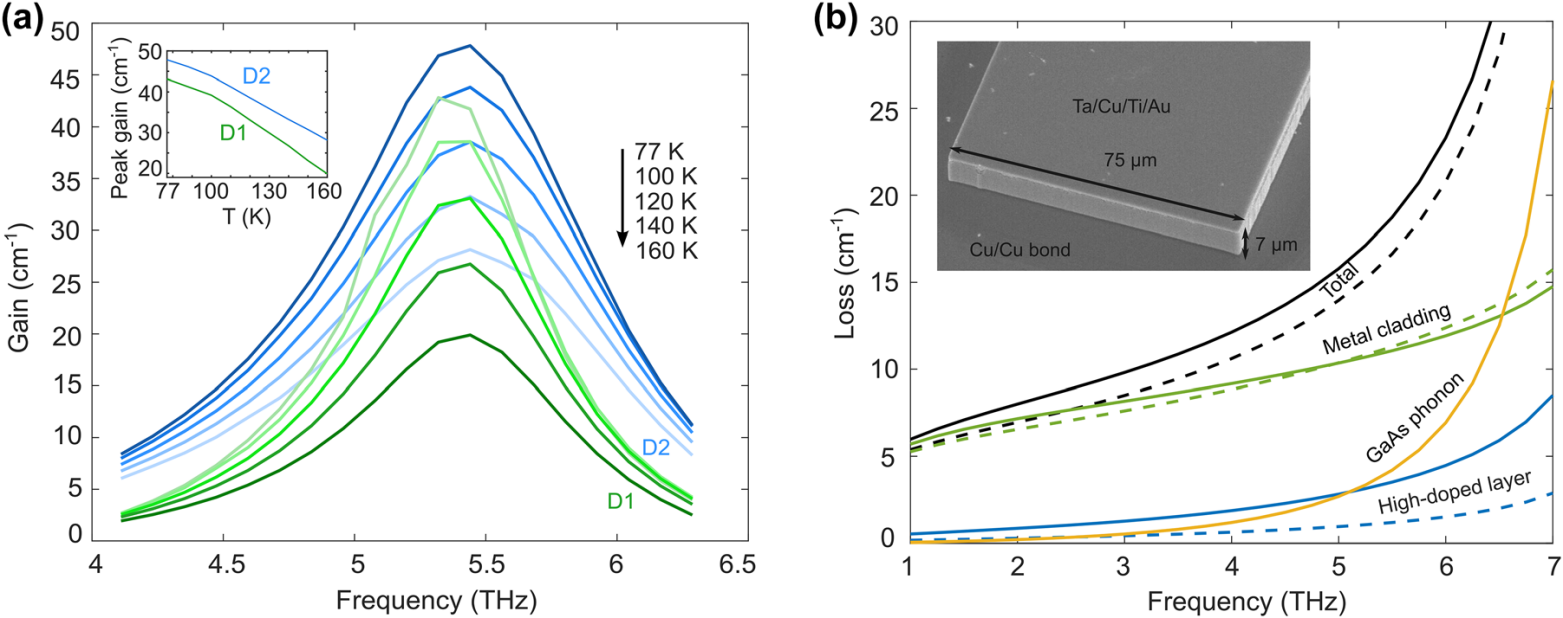
Simulated active region gain spectra and waveguide loss coefficient. (a) NEGF simulation of the gain spectra for various temperatures for design 1 (green) and design 2 (blue). The inset is a plot of the peak gain for D1 and D2 versus temperature. (b) COMSOL simulation of different components of the MM waveguide loss coefficient versus frequency, for a 75 µm wide and 7 µm thick ridge. Solid lines correspond to the first fabrication (Ti/Au top contact), and dashed lines correspond to the second fabrication (Ta/Cu/Ti/Au top contact, and the top high-doped contact layer removed). The inset shows the SEM of the fabricated MM waveguide.

Using these strategies, we chose to proceed with two designs for experiments, as shown in Figure 1. The first design D1 is similar to a design previously tested in our group at 4.7 THz with the same injection and radiative barrier thicknesses (see Supplementary Material, Figure S1). The wells, however, are incrementally adjusted to scale the lasing frequency to 5.4 THz. The second design D2 is optimized using the previously discussed strategies informed by the NEGF simulations. The key design parameters for both designs are summarized in Table 1, and the layer sequences are listed in the Methods section. A radiative barrier of 17 Å is chosen for D2, which reduces *f*_54_ and *f*_53_. To counter the reduced oscillator strength, a sheet doping density of 7.4 × 10^10^ cm^−2^ is used. The downside of the higher doping density is the inevitable increase in the *J*_max_ and the difficulty in achieving cw operation. Thus, a thicker injection barrier of 40 Å is chosen to slightly reduce *J*_max_. Next, thinner well widths are chosen for D2 to increase the upper-to-parasitic energy separation (*E*_75_) to 62 meV and reduce scattering to parasitic states. Doing so, *E*_21_ is also increased to 48 meV, which reduces the ISB losses. The simulated gain spectra for both designs are shown in Figure 3(a). It is immediately apparent that the gain spectra of D2 have higher peak gain values and broader linewidth. Additionally, the peak gain of D2 drops at a slower rate with temperature than D1 (inset of Figure 3(a)).

**Table 1:** Summary of key design parameters and experimental results.

Design	*E*_54_ (meV)	*f*_54_, *f*_53_	Δ_0_ (meV)	*E*_75_ (meV)	*E*_21_ (meV)	*J*_max_ (45 K) (A cm^−2^)	(*J*_max_- *J*_th_)/*J*_max_ (45 K)	Pulsed *T*_max_ (K)	cw *T*_max_ (K)	Radiative/injection barrier (Å)	Sheet doping density (cm^−2^)
1	22.5	0.14, 0.15	1.33	49	41.6	314	0.63	83	68	14/37	3 × 10^10^
2	23	0.12, 0.14	1.35	62	48.1	1124	0.67	117	68	17/40	7.4 × 10^10^

Note: *E*_54_ is the energy spacing between upper and lower state, *f* is the oscillator strength of the transition, Δ_0_ is the anticrossing gap (*E*_1’5_ at design bias), *E*_75_ is the energy spacing between the upper state and parasitic state, *J*_max_ and *J*_th_ are the experimental maximum current density and threshold current density (at 45 K, pulsed), respectively, and *T*_max_ is the experimental maximum operating temperature. Design 1 is from wafer no. VB1400, and design 2 is wafer no. VB1401.

## 3 Device fabrication and testing

Both D1 and D2 structures were grown using molecular beam epitaxy (MBE) growth on GaAs substrates and fabricated into metal–metal (MM) Fabry–Pérot ridge waveguides using Cu–Cu thermocompression bonding process [20], [21] followed by substrate removal and photolithographic definition and dry etching using a self-aligned metal mask (see Section 5). An example SEM of a fabricated ridge with dry-etched facet is shown in Figure 3(b).

Two fabrication runs were performed on these wafers. In the first fabrication, the top 100 nm-thick *n* ^+^ GaAs layer was left un-etched to minimize the parasitic voltage drop at the top Ti/Au metallic contact. In general, the ridge-waveguide lasers tested from this fabrication run (1 mm × 75 µm) showed poor performance. Design 1 did not lase at all when tested down to 40 K, and design 2 lased with a *T*_max_ of 67 K, which is much lower than we expected from the simulations. We attribute this poor performance to excessive waveguide loss. This is justified by the simulated waveguide losses for these waveguides shown in Figure 3(b) (details of the simulation in Section 5). The main contributors to the total waveguide loss are the metal cladding, high-doped layer, and GaAs phonon losses, all of which increase strongly with frequency. It is noted that while the loss from the heavily doped contact layers is not significant at frequencies below 4 THz, its contribution increases strongly at higher frequencies as it approaches GaAs plasma frequency (21.6 THz for 5 × 10^18^ cm^−3^ doping). While removing the lower heavily doped layer was not possible once the wafer was grown, we refabricated MM ridge waveguides in which the upper heavily doped contact layer was etched away; the improvement is shown in Figure 3(b) (dashed blue line). Additionally, the top metal contact was changed to Ta/Cu/Ti/Au since improvements in THz QCL temperature performance have been observed by using Cu waveguides instead of Au waveguides [22]. Because the appropriate material parameters for thin metal films at low temperature are uncertain, our simulations are ambiguous on this point – not much improvement is predicted by switching to Ta/Cu below 5 THz, and the new metal stack can be slightly more lossy above 5 THz (dashed green line). Therefore, the potential improvement from the new metal stack is debatable. This new waveguide geometry reduces the waveguide loss by around 2.1 cm^−1^ at 5.4 THz (dashed back line). An additional improvement is achieved by testing a longer ridge, as the facet reflectance for MM waveguides gets smaller for higher frequencies [23], [24]. Full-wave simulations show that the facet reflectance is 0.57 at 5.4 THz for a 75 µm wide, 7 µm thick MM waveguide with dry-etched facets. Therefore, an additional 2.8 cm^−1^ reduction in loss is achieved by testing a 2 mm long ridge instead of 1 mm. Therefore, we expect an overall 4.9 cm^−1^ reduction for the second round of devices.

**Figure 4:**
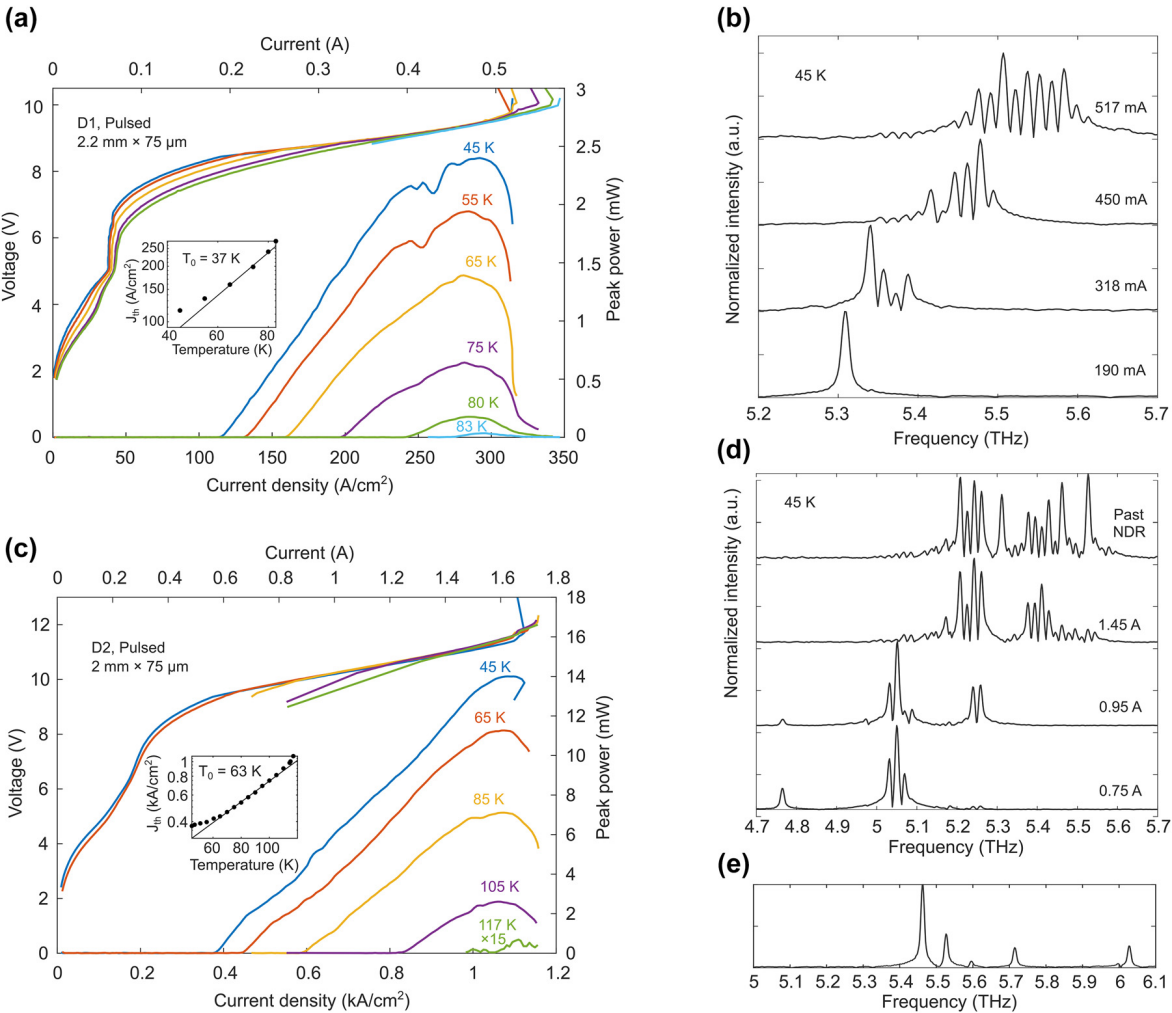
Pulsed *L*–*I*–*V* data and spectra (at 45 K) of MM waveguide for (a, b) design 1 (2.2 mm × 75 μm) and (c, d) design 2 (2 mm × 75 μm). The inset of the *L*–*I*–*V* figures shows the *T*_0_ parameter fitting, and (e) shows the spectrum for a shorter ridge (0.5 mm) from design 2 with modes up to 6.03 THz at 45 K.

**Figure 5:**
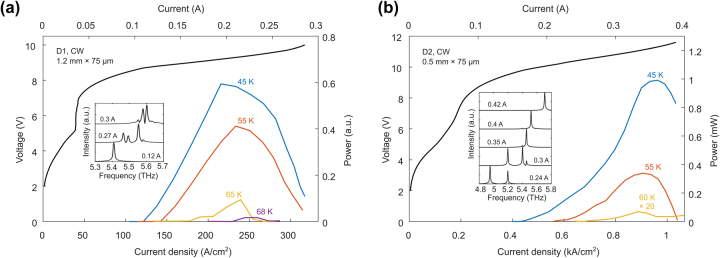
Continuous-wave *L*–*I*–*V* data and spectra for (a) design 1 and (b) design 2. The insets show the corresponding cw spectra at 45 K.

The pulsed light–current–voltage (*L*–*I*–*V*) and spectra of both designs are shown in Figure 4. The ridge from D1 lased pulsed with a *T*_max_ of 83 K with modes from 5.31 to 5.61 THz (at 45 K). The ridges from D2 gave a higher pulsed *T*_max_ of 117 K with modes spanning 4.76–6.03 THz (at 45 K). The characteristic temperature *T*_0_ is extracted for both devices using the empirical relation for threshold current density versus temperature (*J*_th_ = *J*_0_ exp (*T*/*T*_0_)), as shown in the inset of Figure 4(a) and (c), and they are 37 K and 63 K for D1 and D2, respectively. The higher *T*_0_ of D2 is presumably a result of reduced upper state carrier scattering due to a larger *E*_75_ and a more diagonal transition. Additionally, D2 has a higher peak power and dynamic range than D1. These devices also lased in cw mode, as shown in Figure 5, with a *T*_max_ of 68 K and 60 K for D1 and D2, respectively. The cw spectra of D2 spans 4.95–5.71 THz (at 45 K), while the spectra of D1 has modes from 5.4 to 5.6 THz. Although D2 has a higher pulsed *T*_max_, the cw *T*_max_ of D1 is higher in this case. This is because of the higher current level and power dissipated by D2 device; in fact, a narrower ridge from D2 (15 µm wide) increased the cw *T*_max_ of D2 to 68 K due to a more favorable thermal geometry and reduced power dissipation (see Supplementary Material, Figure S2). So far, the cw *T*_max_ of D1 and D2 are tied at 68 K, even though D2 has a higher pulsed *T*_max_. This can be explained by noting that the power density for D1 is 4.4 MW/cm^3^, which is ∼4 times smaller than that of D2. This almost matches the ratio of Δ*T*_D2_/Δ*T*_D1_ ∼ 3.3, where Δ*T* is the difference between pulsed and cw *T*_max_. This clearly demonstrates the importance of keeping the device’s power density low for cw operation, and that the strategy for achieving high operating temperature in pulsed mode does not necessarily hold true for cw mode, unless it is paired with an effective waveguide design. For example, one way to mitigate this may be to improve heat removal from the sidewalls by using buried heterostructures [25], [26], although this makes the fabrication quite challenging. Alternatively, thinner active regions can be considered to improve heat extraction [27]. While this approach will slightly increase the waveguide loss and reduce the pulsed *T*_max_, it may be an effective way to reduce Δ*T* and increase the cw *T*_max_ above the liquid nitrogen temperature (77 K).

## 4 Conclusions

In summary, we have proposed and demonstrated strategies for improving the operating temperature of THz QCLs above 5 THz. Furthermore, we identified the main source of losses for MM waveguides above 5 THz. Employing these design strategies and by improving the waveguide design, we have demonstrated ridge THz QCLs that emit in pulsed mode up to 6.03 THz and in cw mode up to 5.71 THz and achieved *T*_max_ of 117 K in pulsed mode and 68 K in cw mode. This is the highest reported frequency and *T*_max_ above 5 THz to date for a THz QCL, in both pulsed and cw mode. The relatively high power and broad spectra of D2 make this active region a suitable choice for the development of local oscillators in the 5–6 THz range. The devices reported here have not been optimized for output power or beam pattern – both can be improved by implementation of this active region in an end-fire antenna cavity [3] or a metasurface vertical-external-cavity surface-emitting-laser (VECSEL) configuration [28]. The fact that we observed lasing at various modes ranging over 4.76–6.03 THz from a single active region suggests that broadband tuning is also feasible in a tunable VECSEL [29]. Given the robust performance of these devices, and informed by NEGF numerical design optimization, it is likely that THz QCL operation above 6 THz is possible.

## 5 Methods

### 5.1 Active region transport modeling

The modeling of the 4-well BTC-RP design was done using a Schrödinger solver, and then the electron dynamics and gain spectra were investigated using the nextnano simulation package, which is based on an NEGF solver [15]. The NEGF model accounts for both coherent transport effects such as resonant tunneling and incoherent evolution such as scattering mechanisms (namely electron–electron, impurity, interface roughness, acoustic, and LO phonon scattering). We simulate two modules of the active region to include the full effect of coherence and tunneling for both laser states and the higher-lying channels. The energy range for the calculations is selected to be as large as the conduction band offset to account for all the high-level states. For the GaAs/AlGaAs system, we consider electron transport from carriers in the Γ-valley with an effective mass of 0.067*m*_0_, where *m*_0_ is the free electron mass. The nonparabolicity is accounted for using a 3-band model. The electron-optical-phonons are mediated through the Fröhlich interaction, and a LO phonon energy of 36 meV is used for GaAs. The NEGF simulations naturally include ISB gain/loss from all possible level pairs (especially 1 → 2); therefore, no active-region free-carrier loss is explicitly included in the waveguide simulations. Finally, although the dispersive effects of the phonon band are included in the material index, GaAs bulk loss is excluded from the model, so that it can be separately included in the waveguide loss model.

### 5.2 Finite element method simulation

The waveguide loss simulations are performed using COMSOL Multiphysics 6.1, using the electromagnetic wave, frequency domain (emw) interface in the radio frequency module. The MM waveguide is simulated in a two-dimensional cross section eigenmode solver and includes all the layers of the device as-fabricated: bottom metal contact: Ta/Cu, top and bottom high-doped GaAs layers, active region, and top metal contact: Ti/Au, and Ta/Cu/Ti/Au. The permittivity of the metallic and doped layers is fitted using the Drude model, and the Drude parameters are taken from [30]–[33], and they are listed in the supplementary material (Table S1). The loss is extracted from the imaginary part of the mode index.

### 5.3 QCL growth and MM waveguide fabrication/characterization

The QCL active layer is grown on a GaAs wafer using molecular beam epitaxy. The growth sequence for the two devices used in this work is listed below. The layer thicknesses are given in angstroms, the Al_0.25_Ga_0.75_As barriers are in boldface, and the Si-doped layers are underlined. Design 1: 93/**14**/112/**28**/85/**31**/161/**37**, where the middle 59 Å of the underlines layer is doped at 5 × 10^16^ cm^−3^ (wafer No. VB1400). Design 2: 86/**17**/97/**28**/75/**31**/147/**40**, where the entire underlined layer is doped at 5 × 10^16^ cm^−3^ (wafer No. VB1401). The growth starts with a GaAs buffer layer followed by a 200 nm Al_0.55_Ga_0.45_As etch-stop layer and a 100 nm high-doped GaAs layer (5 × 10^18^ cm^−3^). A total of 118 and 127 repetitions of QCL stages (GaAs/Al_0.25_Ga_0.75_As) are grown for D1 and D2, respectively. The growth is then followed by 50 nm of high-doped GaAs layer (5 × 10^18^ cm^−3^) as well as 10 nm very highly doped GaAs layer (5 × 10^19^ cm^−3^) and a low-temperature grown GaAs cap layer. Total epitaxial thickness is 7 µm. The fabrication starts by Ta/Cu (10/300 nm) evaporation on the epitaxial wafer piece and a receptor GaAs wafer. The pieces are then bonded in vacuum by thermocompression bonding at 350 °C for 1 h and followed by 1 h of anneal time at the same temperature. Next, the substrate of the epitaxial pieces is mechanically lapped until ∼50 µm remains, and the rest is removed using a citric acid selective wet etch. The Al_0.55_Ga_0.45_As etch stop layer is then etched by a few seconds dipping in hydrofluoric acid solution. Next, the 100-nm-thick heavily doped GaAs layer is removed by wet-etching. Top metal contact, either Ti/Au/Ni (15/250/200 nm) or Ta/Cu/Ti/Au/Ni (10/135/20/150/200 nm), is then defined using photolithography and is used as a self-aligned mask to etch the active region with a BCl_3_/Cl_2_ ICP-RIE dry etch. The etch is stopped at the bottom copper layer to enable direct wire bonding to the ground plane. The Ni etch mask is then chemically removed, leaving behind the exposed Au surface. Finally, the back contact metallization Ti/Au (15/300 nm) is evaporated on the backside of the wafer piece. After fabrication is complete, laser ridges are cleaved and mounted on copper submount using indium bonding, and then they are wire bonded for characterization. The bottom contact pad is directly wire bonded to the exposed ground plane to minimize any parasitic voltage drops. The ridges are then characterized by mounting on a cold finger of a Stirling cycle cryocooler (Longwave Photonics). In pulsed mode, the ridges are biased with 500-ns pulse width and 100 kHz repetition rate, and the power is measured using a pyroelectric detector (Gentec). The absolute power is measured with a calibrated thermopile detector (Scientech). The spectra are measured with a Nicolet FTIR in continuous-scan mode, with the optical path purged by nitrogen gas.

## Supplementary Material

Supplementary Material Details
