# Supplementary material for: Continuous-wave GaAs/AlGaAs quantum cascade laser at 5.7 THz
Source: Nanophotonics. 2024 Jan 16;13(10):1735–43. doi: 10.1515/nanoph-2023-0726 (PMC11052532; doi:10.1515/nanoph-2023-0726)
Supplement: Supplementary file 1 — Supplementary Material Details [file j_nanoph-2023-0726_suppl_001.docx]

**Supplementary material for**

**Continuous-wave GaAs/AlGaAs quantum cascade laser at 5.7 THz**

Mohammad Shahili^1^, Sadhvikas J. Addamane^2^, Anthony D. Kim^1^_,_ Christopher A. Curwen^3^, Jonathan H. Kawamura^3^, and Benjamin S. Williams^1^

*^1^Department of Electrical and Computer Engineering, University of California, Los Angeles, California 90095, USA.*

^2^*Sandia National Laboratories, Center of Integrated Nanotechnologies, MS 1303, Albuquerque, New Mexico 87185, USA.*

*^3^Jet Propulsion Laboratory, California Institute of Technology, Pasadena, California 91109, USA.*

1. **4.7 THz device**

The D1 design in the main manuscript is an incremental change of a design previously tested aimed at 4.7 THz. Some fundamental characteristics of that design are presented here. The active layer is 5 µm thick and is based upon the GaAs/Al_0.25_Ga_0.75_As composition. The layer thicknesses are given here in angstroms, where the barriers are bolded: 95/**14**/110/**28**/85/**31**/162/**37.** The middle 88 Å of the underlined layer is doped at 5 × 10^16^ cm^-3^ to give a sheet density of 4.4 × 10^10^ cm^-2^ per module (wafer No. VB1247). The pulsed light-current-voltage (*L-I-V*) data and spectra for a metal-metal (MM) waveguide with dry-etched facets for the 4.7 THz device is shown in Figure S1. It has a pulsed and continuous wave (cw) maximum operating temperature (*T*_max_) of 130 K and 93 K, respectively.

**Fig. S1:** (a) Pulsed L-I-V data of metal-metal waveguide (1 mm × 50 μm) for the 4.7 THz device (b) Corresponding spectra at 77 K for various bias points.


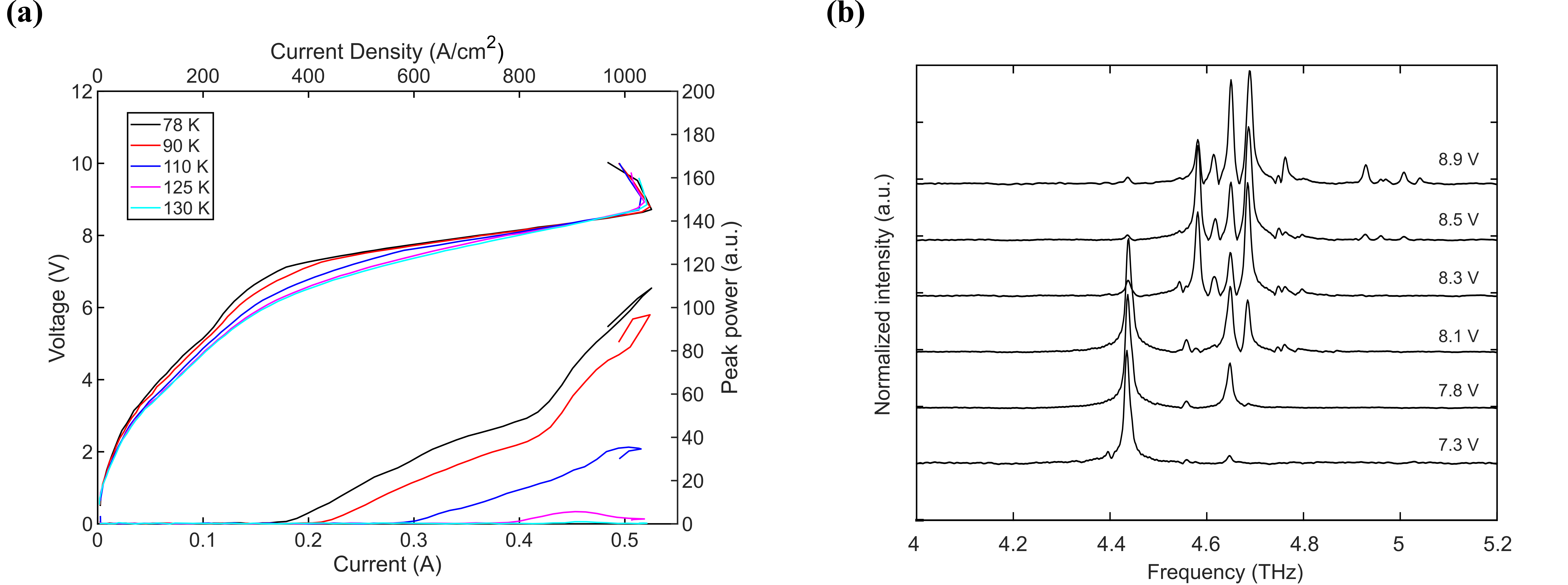


1. **Characterization of Design 2, in a narrow ridge device**

To evaluate whether a narrower ridge waveguide could increase the cw *T*_max_ of design 2 (D2), we fabricated 15-µm-wide ridges from the same wafer (No. VB1401) using the same fabrication steps that were used to make the previous MM waveguide ridges, with several changes made to allow these devices to share a process run with other wafers. First, we used Au-Au thermocompression bonding instead of Cu-Cu, which means that the lower waveguide metallization is Ta/Au. Au-Au bonding was done to avoid the possibility of oxidation associated with a copper ground plane, and allow wire bonding directly to the ground plane. Second, we used a Ti/Au top metal contact instead of Ta/Au, which reduced the likelihood of photoresist burning during the Ta deposition. Third, the ridges have dedicated wire bonding pads that are connected to the ridge using a narrow connector. These pads have an insulating layer of SiO_2_, so that they will not significantly add to the current draw of the device. A cartoon of the fabricated ridge (not to scale) is shown in the inset of Figure S2(a).

**Fig. S2:** Pulsed (a,b) and continuous-wave (c,d) L-I-V and spectra of metal-metal waveguide for a narrow ridge, design 2. The inset in (a) shows a cartoon of the ridge with the wirebond pad (not to scale).


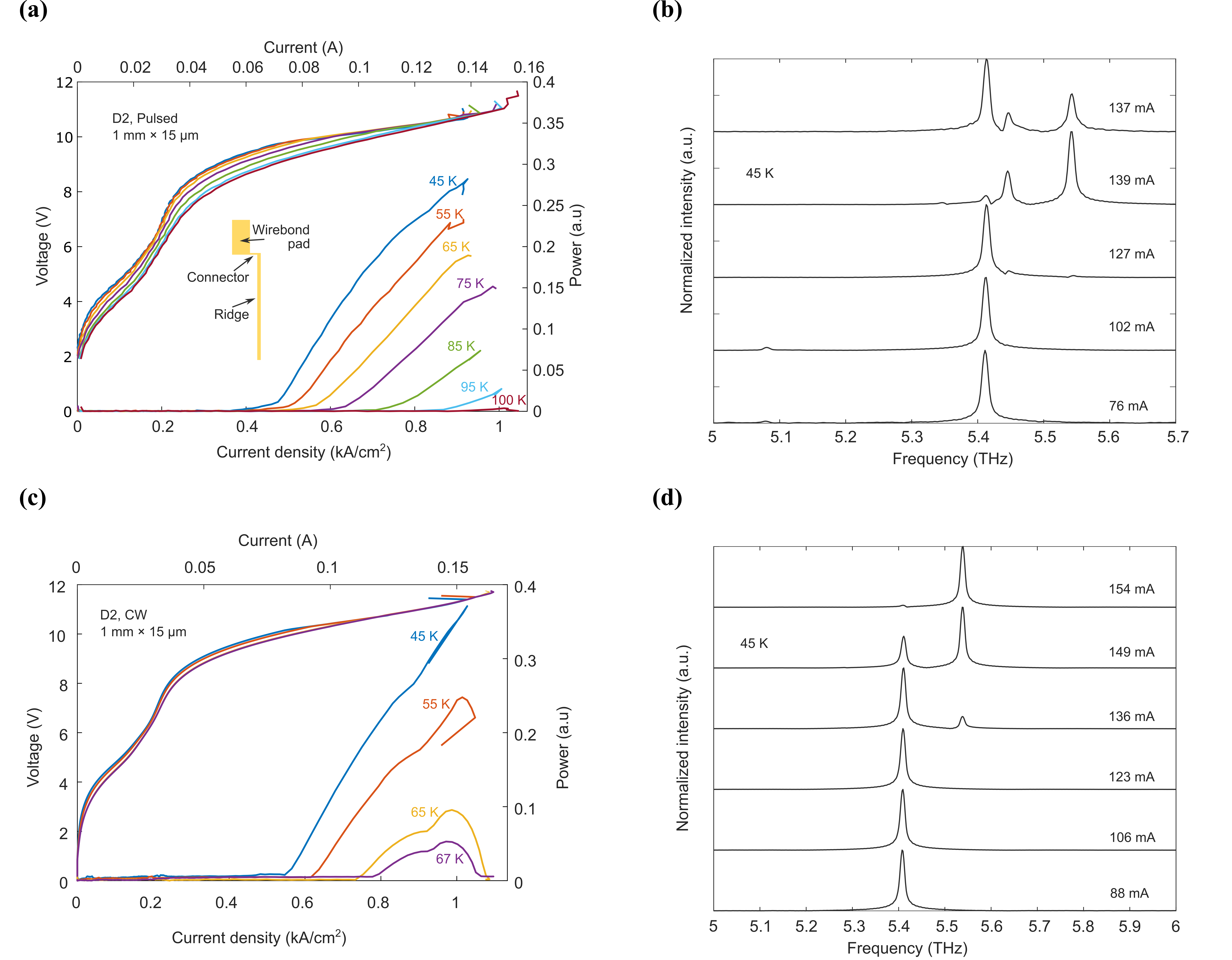


The pulsed and cw *L-I-V* and the corresponding spectra are shown in Figure S2. The pulsed *T*_max_ was 100 K, somewhat reduced from the value of 117 K for the 2 mm × 75 μm device in the main manuscript. However, due to the reduced power consumption and more favorable thermal geometry, the cw *T*_max_ is 68 K, compared to 60 K of the wider device. The reduction in the pulsed *T*_max_ is somewhat larger than expected. We propose several reasons for the reduction in pulsed *T*_max_. First, it is possible that the presence of the contact connector has changed the shape of the dry etched facet and created additional facet loss. Second, a COMSOL simulation indicates the 15-μm-wide waveguide has a slightly lower modal confinement factor Γ of 96%, and slightly higher waveguide loss of *α*_w_=21.9 cm^-1^ (compared with 99.9% and 21.2 cm^-1^ respectively for the 75-µm-wide ridge) (see Figure S3). This would increase the threshold gain (*g*_tr_= *α*_w_/Γ) by about 8%, which would reduce *T*_max_ accordingly. Third, the mode in the narrower ridge is also stronger at the ridge sidewalls, which means that it is more sensitive to any sidewall roughness introduced during the fabrication. Therefore, it seems that there is the trade-off between reducing device’s power consumption and having higher confinement factor and lower waveguide losses. It is likely an optimized device could be engineered to operate in cw mode above 77 K.


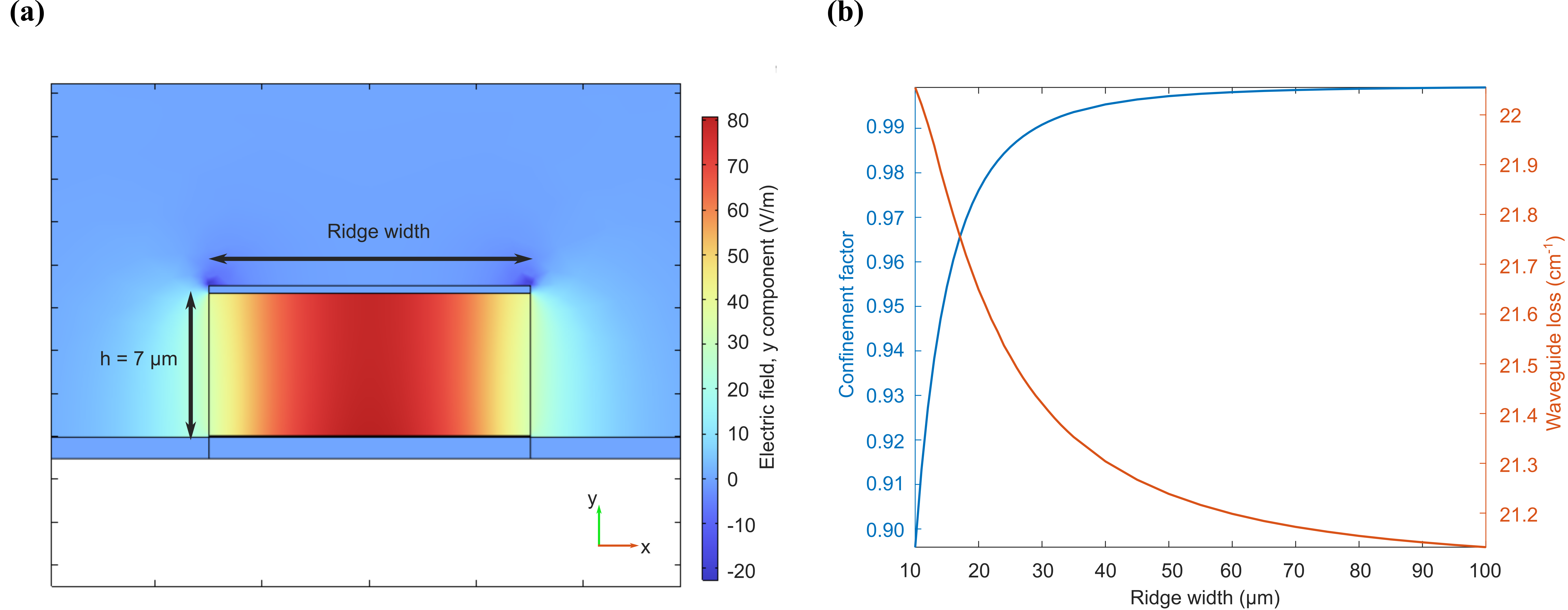


**Fig. S3:** (a) FEM simulation of the fundamental mode (E_y_) of the metal-metal waveguide at 5.4 THz with Ta/Au bottom and Ti/Au top metal contacts. (b) Confinement factor of the fundamental mode and the waveguide loss at 5.4 THz versus ridge width for a 7-μm-high ridge.

1. **Drude material parameters for electromagnetic finite element simulations**

The Drude parameters that are used in the COMSOL simulations are listed in Table S1, which are taken from [S1–S4].

| **Material** | ***ω_p_*/2π (PHz)** | ***τ* (fs)** |
| --- | --- | --- |
| Tantalum (Ta)^*^ | 1.98 | 7.5 |
| Copper (Cu) ^*^ | 1.79 | 73 |
| Titanium (Ti) ^*^ | 0.627 | 22 |
| Gold (Au) ^**^ | 2.18 | 39 |
| n+ GaAs ^***^ | 0.0216 | 90 |

**Table S1:** Summary of Drude parameters used in the finite-element method (COMSOL) simulation.

Note: *ω*_p_ is the plasma frequency. τ is the Drude scattering lifetime.

* Parameters taken from [S1-S2]

** Parameters taken from [S3]

*** Parameter taken from [S4]

**References**

[S1] M. A. Ordal, R. J. Bell, R. W. Alexander, L. L. Long, and M. R. Querry, “Optical properties of fourteen metals in the infrared and far infrared: Al, Co, Cu, Au, Fe, Pb, Mo, Ni, Pd, Pt, Ag, Ti, V, and W,” *Appl. Opt.*, vol. 24, no. 24, p. 4493, 1985, <https://doi.org/10.1364/AO.24.004493>.

[S2] M. A. Ordal, R. J. Bell, R. W. Alexander, L. A. Newquist, and M. R. Querry, “Optical properties of Al, Fe, Ti, Ta, W, and Mo at submillimeter wavelengths,” *Appl. Opt.*, vol. 27, no. 6, p. 1203, 1988, <https://doi.org/10.1364/AO.27.001203>.

[S3] N. Laman and D. Grischkowsky, “Terahertz conductivity of thin metal films,” *Applied Physics Letters*, vol. 93, no. 5, p. 051105, 2008, <https://doi.org/10.1063/1.2968308>.

[S4] J. Lloyd-Hughes, Y. L. Delley, G. Scalari, et al., “Spectroscopic determination of the doping and mobility of terahertz quantum cascade structures,” *Journal of Applied Physics*, vol. 106, no. 9, p. 093104, 2009, <https://doi.org/10.1063/1.3247973>.
